# Ineffectiveness of Reverse Wording of Questionnaire Items: Let’s Learn from Cows in the Rain

**DOI:** 10.1371/journal.pone.0068967

**Published:** 2013-07-31

**Authors:** Eric van Sonderen, Robbert Sanderman, James C. Coyne

**Affiliations:** 1 Department of Health Sciences, Section Health Psychology, University of Groningen, University Medical Center Groningen, Groningen, The Netherlands; 2 Department of Psychiatry, Perelman School of Medicine, University of Pennsylvania, Philadelphia, Pennsylvania, United States of America; Iran University of Medical Sciences, Iran (Republic of Islamic)

## Abstract

**Objective:**

We examined the effectiveness of reverse worded items as a means of reducing or preventing response bias. We first distinguished between several types of response bias that are often confused in literature. We next developed arguments why reversing items is probably never a good way to address response bias. We proposed testing whether reverse wording affects response bias with item-level data from the Multidimensional Fatigue Inventory (MFI-20), an instrument that contains reversed worded items.

**Methods:**

With data from 700 respondents, we compared scores on items that were similar with respect either to content or to direction of wording. Psychometric properties of sets of these items worded in the same direction were compared with sets consisting of both straightforward and reversed worded items.

**Results:**

We did not find evidence that ten reverse-worded items prevented response bias. Instead, the data suggest scores were contaminated by respondent inattention and confusion.

**Conclusions:**

Using twenty items, balanced for scoring direction, to assess fatigue did not prevent respondents from inattentive or acquiescent answering. Rather, fewer mistakes are made with a 10-item instrument with items posed in the same direction. Such a format is preferable for both epidemiological and clinical studies.

## Introduction

The use of both positively and negatively worded items in questionnaires was introduced decades ago in order to prevent response bias. Response bias refers to answer patterns on questionnaires that do not reflect the respondents’ actual state or opinion [1], and that thus can pose a serious threat to the validity of self-report instruments [2].

We have three goals with this paper. First, we want to distinguish among several types of response bias that are confused in literature. Next, we develop arguments why reversing items is probably almost never a good way to prevent or deal with response bias. Finally, as an example, we tested reverse wording of items with data from the Multidimensional Fatigue Inventory (MFI-20), an instrument on which ten of the items are reverse worded.

### Response Bias

The process of obtaining survey data is complex, with many possibilities of discrepancies arising between the state or opinion the researcher wants to elicit and the answer given by the respondent [3]. Usually this discrepancy is called response bias [4]. Based on Rorer [1], Weijters [5] distinguishes two main types of response bias: response set and response style. He defines *response set* as bias related to the content of the items and *response style* as a tendency to answer items regardless their content. The best known type of response set is social desirability, in which a person’s response is a function of the desirability of the response rather than its veracity. Three types of response styles can be distinguished.

Respondents may have read and understood completely the question and answer categories, but nonetheless be inclined to agree with statements in general (acquiescence), to disagree (disacquiescence), or to give extreme answers, or, alternatively, less extreme answers. Consistent with Swain et al. [6], we will take *acquiescence* as an example of this type of response styles.

Respondents may also lack sufficient attention to carefully read both the question and answer categories, and thus, by missing the intended meaning of an item, give a response that may differ from the true value. Krosnick [7] mentions a satisficing response style, whereby the respondent deliberately makes less effort to understand all subtleties of the question. We will call this style *inattention,* irrespective whether the respondent is aware of it or not.

Finally, the question in combination with answer categories may be too difficult for a respondent to comprehend. Sometimes the respondent may think the item is well understood, but still an error can be made, due to a high level of difficulty [6]. The respondent may however be aware of this difficulty, and thus the answer can be considered a ‘best guess’. We will call both varieties of this type *confusion*.

According to Weijters et al. [8] we focus on response style in discussing the consequences of reversed worded items. We consider different types of response set, like social desirability, to be less sensitive for reversed worded items.

It is the challenge for a researcher to deal with all these threats and still obtain an optimal answer, that is, one that consistently resembles the true value.

### Reversing Items

Reversing a portion of the items is often intended to reduce the effects of response styles, although there is no consensus that this is an effective strategy. In general, two strategies are available for item reversal [6]. The first consists of adding negative particles: words like ‘not’ or ‘no’ or affixal morphemes like ‘un-‘, ‘non-‘, ‘dis-‘ or ‘-less’. In this case the direction of the item is changed without changing substantially item wording. The new item is considered to be ‘*reverse oriented*’. The second strategy is using words with an opposite meaning. For example, the opposite of “I feel fit” is “I feel fatigue”. In this case the direction of the new item is changed by means of ‘*reverse wording*’. Swain et al. [6] analyzed nearly 2000 items and found that 81% of the reversed items were negations, i.e. items created by the first strategy.

We will now discuss the consequences of item reversal in the light of the three response styles we have introduced, acquiescence, inattention, and confusion.

### Reversal and Acquiescence

Although many researchers advocate the use of reversed items in order to address acquiescence, it is doubtful whether this response style will be affected by the direction in which the items are formulated. Considering acquiescent respondents as people who carefully read each question, they will, when confronted with a reversed item, still agree, and thus leave the researcher with an uninterpretable patterning of answers (someone who is both ‘tired’ and ‘fit’). If a respondent agrees with an ordinary item and disagrees with a reversed worded item, according to the true state, then by definition this person is not an acquiescent respondent from whom we expect only affirmative answers on all items.

### Reversal and Inattention

Several types of inattention can be distinguished. First, a respondent may miss the presence of a negative particle or an affixal morphem, for example s/he may read ‘I am healthy’ instead of ‘I am not healthy’ or ‘I am unhealthy’. This type of inattention relates to any individual item. A respondent may also miss the fact that a consecutive item is formulated in a reversed way, compared to the previous one. Finally, a respondent can miss the difference in content between two consecutive items.

As Drolet and Morrison [9] demonstrate, respondents sometimes just answer the first item and assume that this answer also holds for subsequent (considered similar or even identical) items. Sometimes respondents do not bother to endorse an answer for each item individually, but draw one large circle around the same response for all items. The risk for this type of inattention grows with the extent to which items resemble each other, and when scales are longer. Only the second type of inattention, missing subtle differences with respect to content, can be challenged by reversing some items, provided that respondents are not inattentive to the reversal, in which case reversing will be counterproductive.

### Reversal and Confusion

The last response style that has to be considered when reversing items, is confusion. As Swain et al. [6] demonstrated, item verification difficulty, that is respondents’ difficulty interpreting items, increases when reverse oriented items rely on negative particles or affixal morphemes. Modifying an item by reverse wording, and thus inquiring about the opposite state, will only lead to more difficulties in interpretation, if the described state is not in accordance with the respondents actual state. For example, for a tired person, the item ‘I am tired’ is easy, and ‘I am fit’ is a bit more difficult since this item has to be denied. For a fit person however, the opposite holds.

### Demonstrating or Preventing Response Styles

With these arguments in mind it does not seem advisable in scale construction to reverse a portion of the items. Schriesheim and Hill [10] previously concluded that reversed worded items, when used in an effort to control for acquiescence, lowered questionnaires’ validity. Yet, many instrument developers persist in adhering to this strategy. Usually their intention is to assess a one-dimensional construct and reversing some items is seen as limiting the influence of response styles, especially acquiescence. Yet, the unintended consequence is the emergence of two factors in subsequent factor analyses, commonly precipitating a debate in the literature whether or not two meaningful concepts can be distinguished where only one was intended. Eventually papers are published concluding that the second factor is just a methodological artifact, caused by the use of reversed worded items. We mention some examples:

1. Meyer and Allen’s [11] scale to assess affective commitment, of which Merritt [12] demonstrated that the answers on reversed items were prone to careless responding and cognitive fatigue.
2. Roszkowski and Soven [13] examined a questionnaire used in student evaluations, that contained two reversed worded items among mainly positively worded items, and concluded that replacing the reversed worded items by ordinary items improved the internal consistency
3. Meyer et al.’s [14] scale to assess worry contains 11 positively and five reversed worded items, leading Fresco et al. [15] to the conclusion of two distinct concepts, Worry Engagement and Absence of Worry. Hazlett-Stevens et al. [16] however conclude that the reversed worded items caused a method factor.
4. Bradley et al. [17] inspected the behavior of ten items from a scale used to ask students to evaluate courses and instructors. This questionnaire contains five pairs of items that were worded in opposite directions (e.g. “the professor was unprepared for class” versus “the professor was prepared for class”). They concluded that the use of opposite items introduced noise, known as measurement error.

Summarizing, we see no convincing arguments that reversing part of the items will prevent response styles of any kind. When no items are reversed, the answer pattern of an acquiescent person cannot be distinguished from the pattern of a person who intends to agree with all the items. Confronting some reversed items, the acquiescent person will make mistakes that can thus be detected. Unfortunately, the opposite does not hold: agreeing with all items, including the reversed ones, may not only be caused by acquiescence, but also by inattention or by confusion. Hinz et al. reported that an acquiescence response style can be detected in instruments with some reverse worded items [18]. Although they stated that in their sample 6% of the respondents on the MFI-20, showed ‘pronounced acquiescence’, they also admitted that this ‘acquiescence’ could be due to ‘differences in the verbal diction of the items’. Woods [19] found that carelessness among 10% of the respondents on a scale containing reversed worded items, would already lead to an artificially created factor, inducing researchers to erroneously reject unidimensionality. More researchers attribute the claim of multidimensionality that is often made in scales with reversed worded items, not to conceptual differences, but to artificial factors resulting from response bias [20]–[25].

## Methods

### Ethics Statement

Data were collected in a multi-center study from 700 patients with inflammatory bowel disease. Participating centers were Department of Gastroenterology and Hepatology, Maastricht University Medical Center, Department of Internal Medicine and Gastroenterology, Orbis Medical Centre Sittard, and Department of Internal Medicine and Gastroenterology, Atrium Medical Centre Heerlen, all from the Netherlands. The Ethical Committees of all participating centers approved the protocol and written informed consent was obtained from all patients at inclusion in the IBD-SL registry for future analysis and publication of data. In the few patients below 18 years of age their parents or a legal representative signed an informed consent [26].

### Instrument

We will now examine the effect of reversing items on response bias with data collected with the Multidimensional Fatigue Inventory (MFI-20) [27]. The MFI-20 is intended to measure fatigue with twenty items, and consists of five four-item subscales assessing General Fatigue, Physical Fatigue, Reduced Activity, Reduced Motivation, and Mental Fatigue. There is some inconsistency regarding the number of answer categories used in the MFI-20. In 1995 Smets et al. [28] constructed a version with seven categories, but in accordance with their adaptation in 1996 [27], a version is used with five answer categories, ranging from 1 (‘yes, that is true’) to 5 (‘no, that is not true’). The three answer categories in between are lacking a description. Smets et al. [28] used ‘reversed wording’, the second strategy mentioned by Swain et al. [6], to reverse ten of the items, thus measuring fitness. All items measuring one of the five types of fatigue, original or reversed, are presented to the respondent in a mixed order (see table 1).

**Table 1:** MFI-20 items.

a	+	1.	I feel fit
b		2.	Physically I feel only able to do a little
c	+	3.	I feel very active
d	+	4.	I feel like doing all sorts of nice things
a		5.	I feel tired
c	+	6.	I think I do a lot in a day
e	+	7.	When I am doing something, I can keep my thoughts on it
b	+	8.	Physically I can take on a lot
d		9.	I dread having to do things
c		10.	I think I do very little in a day
e	+	11.	I can concentrate well
a	+	12.	I am rested
e		13.	It takes a lot of effort to concentrate on things
b		14.	Physically I feel I am in a bad condition
d	+	15.	I have a lot of plans
a		16.	I tire easily
c		17.	I get little done
d		18.	I don't feel like doing anything
e		19.	My thoughts easily wander
b	+	20.	Physically I feel I am in an excellent condition

Codes preceding item numbers, referring to subscales:

a general fatigue.

b physical fatigue.

c reduced activity.

d reduced motivation.

e mental fatigue.

+ positively stated.

Answer categories:

yes, that is true.

no, that is not true.

The MFI is intended to assess *fatigue*. Therefore, to avoid misunderstandings, items about *fatigue* are considered to be straightforward, negatively formulated, and items about *fitness* to be the reversed worded, positively formulated, items.

The developers of the MFI explicitly implemented use of reverse worded items to prevent response set, and thus are not seeking to assess two slightly different aspects of fatigue. Although the developers of the MFI-20 state they reversed items to prevent response set [28], it is more likely they intended to prevent response styles. Which of the aforementioned response style(s) they try to prevent remains however unclear.

### Respondents

Data were collected in a multi-center study from 700 patients with inflammatory bowel disease (IBD). The aim of that study was to investigate the prevalence and severity of fatigue and the impact on health-related quality of life in patients included in a population-based IBD cohort in the Netherlands [26]. We are aware of the special characteristics of this sample, and it may not be representative of the general population. We can expect this sample to score higher on fatigue than a healthy sample. However, we are not interested in the average level of fatigue and therefor do not consider any differences with the general population to be a problem in the use of the data set as an example. On the contrary, we wanted to analyze a sample with higher average levels of fatigue, in order to avoid highly skewed distributions of answers.

Respondents were send a questionnaire by post. Of the 707 patients that were asked to fill in the MFI-20, seven had missing data on one or more of the twenty items. For this study, all patients with missing data were deleted. Of the remaining 700 patients, 311 were males with mean age 51.1 years (sd 15.2). The 389 females have a mean age of 44.0 (sd 13.7).

## Results

### Analytic Plan

Each subscale of the MFI-20 consists of four items, two of them reversed worded. First, we will create pairs of original and reversed worded items, that resemble each other maximally with respect to content, except for their direction.

We will then examine the inter-item correlations for each subset of four items. If reversing items reduces response bias, we would expect two items that are identical with respect to content but different in direction, to be stronger related than two items formulated in the same direction, but with a slightly different content. We consider this a variety of the Multi-Trait-Multi-Method matrix discussed by Campbell & Fiske [29].

Although suspicious answer patterns may become visible through lower correlation coefficients, the opposite does not hold. Low correlation coefficients can also occur in a homogeneous sample with small item variances. We will therefor do an additional analyses by checking the percentage of suspicious answer patterns for each pair of items within a subset. We will consider a difference of at least three points to be indicative for a wrong answer on at least one of the two items. The choice for a three points criterion is a bit arbitrary, but still well defensible. Since a 5-point scale is used, there are two options left or right from the middle one, indicating (strong) agreement or (strong) disagreement. We did not want an extreme answer (1 or 5) on one item, together with a 3 on the reversed version, to be considered suspicious. Since this would be a difference of 2 points, we choose a three points criterion, that can only be met by an extreme answer on one item (1 or 5) and an extreme or nearby extreme answer on the opposite item (4 or 5 respectively 1 or 2).

Finally, we will assess the psychometric qualities of the ten items measuring fatigue and the ten items measuring fitness separately. The results will be compared with the scales using both types of items simultaneously. If the assumption holds, that reversing part of the items leads to less response bias, we expect instruments that contain both original and reversed items, to have better psychometric properties than instruments containing only original or reversed items.

The scores on negatively formulated items, i.e. items asking for fatigue, are reversed in order to have higher scores indicating higher levels of fatigue. All analyses were done with SPSS 15.

### Correlations between Pairs of Items

Spearman correlations between pairs of items were computed in order to compare the effect of content with the effect of direction. Results are presented in table 2.

**Table 2:** Spearman correlations between itempairs (item numbers between brackets).

	Same content		Same direction		Differ in content and direction	
General Fatigue	.76	(1–16)	.81	(**5–16**)^1^	.75	(1–5)
	.74	(5–12)	.75	(1–12)	.73	(12–16)
Physical Fatigue	.64	(2–8)	.66	(**2–14**)	.63	(2–20)
	.70	(14–20)	.65	(8–20)	.60	(8–14)
Reduced Activity	.55	(3–17)	.64	(**10–17**)	.42	(3–10)
	.71	(6–10)	.42	(3–6)	.52	(6–17)
Reduced Motivation	.56	(4–18)	.54	(**9–18**)	.43	(4–9)
	.38	(9–15)	.56	(4–15)	.47	(15–18)
Mental Fatigue	.61	(7–19)	.66	(**13–19**)	.62	(7–13)
	.67	(11–13)	.78	(7–11)	.68	(11–19)

^1^In the column ‘Same direction’ bold itempairs refer to negatively formulated items, assessing fatigue.

If reversal of items would have had no effect at all, we would expect the highest correlations between itempairs that resemble each other maximally with respect to content (despite the opposite direction), the pairs in the first column. Also we would expect lower correlations between items that measure (subtle) different aspects of fatigue, irrespective whether this is done with two items formulated in the same direction (column 2) or in opposite directions (column 3).

Overall, the correlations between item pairs formulated in the same direction, shown in the first column, are not consistently higher than in the other columns, indicating an adverse effect of reversed wording.

### Suspicious Answer Patterns

In table 3 percentages of respondents with unlikely combinations of answers are given on pairs of items that (should) measure about the same aspect of fatigue. Again, in column 1 item pairs are presented that resemble each other maximally with respect to content, except for different direction. Ideally we would expect no respondent to give unlikely answers, but if they occur, we would expect them to represent apparently important differences in the content of two related items. Thus, we would expect the highest percentages in column 2 and comparable percentages in column 3. Again the percentages in column 2 are not higher than those in column 1, rather a bit lower, indicating an adverse effect of reversed wording.

**Table 3:** Percentage of large discrepancies (at least three points) between answers on ‘similar’ items.

			Item 8										
			Yes, that is true					No, that is not true					
			1	2	3		4	5					
Item 2													
Yes, that is true	(5)		4*	1*	4		19	57					
	(4)		4*	10	22		37	29					
	(3)		10	14	50		39	19					
	(2)		10	63	44		33	9*					
No, that is not true	(1)		96	74	39		5*	8*					
	Same content			Same direction						Differ in contentand direction			
General Fatigue ^1^	2.0%	(1–16)		2.0%		(**5–16**)^2^			2.4%			(1–5)	
	3.0%	(5–12)		1.9%		(1–12)			2.4%			(12–16)	
Physical Fatigue	4.4%	(2–8)		5.4%		(**2–14**)			10.4%			(2–20)	
	5.7%	(14–20)		5.4%		(8–20)			6.0%			(8–14)	
Reduced Activity	9.9%	(3–17)		3.9%		(**10–17**)			13.9%			(3–10)	
	3.1%	(6–10)		10.6%		(3–6)			5.7%			(6–17)	
Reduced Motivation	5.9%	(4–18)		7.4%		(**9–18**)			8.6%			(4–9)	
	9.0%	(9–15)		5.3%		(4–15)			8.0%			(15–18)	
Mental Fatigue	6.1%	(7–19)		5.1%		(**13–19**)			5.6%			(7–13)	
	4.6%	(11–13)		2.6%		(7–11)			4.0%			(11–19)	

Example: Crosstab of item 2 (Physically I feel only able to do a little) with item 8 (Physically I can take on a lot).

() between brackets are scores after recoding, so that higher scores indicate more fatigue for all items.

* denote cases with a difference of a least three points between items 2 and 8, (4.4%).

^1^Note that the percentages should not be compared rowwise, but by taking the two rows for each type of fatigue together.

^2^In the column ‘Same direction’ bold itempairs refer to negatively formulated items, assessing fatigue.

### Scale Properties for Positive and Negatively Worded Items Separately

Cronbach’s alpha and the mean inter-item correlation were computed for the complete sets of items as well as for the five dimensions of fatigue. This was also done for the positively and negatively worded items separately. Since Cronbach’s alpha is dependent on scale length, smaller alpha’s can be expected when the scale length is reduced by 50%. Results are presented in table 4.

**Table 4:** Cronbach’s alpha (mean inter-item correlation between brackets).

Dimension:	All items	Positive items (fitness)	Negative items (fatigue)
General Fatigue	.93 (.76)	.86 (.76)	.90 (.82)
Physical Fatigue	.88 (.64)	.79 (.65)	.80 (.66)
Reduced Activity	.83 (.54)	.59 (.42)	.78 (.65)
Reduced Motivation	.80 (.49)	.73 (.57)	.70 (.53)
Mental Fatigue	.89 (.66)	.88 (.78)	.79 (.65)
All items	.95 (.47)	.90 (.46)	.91 (.49)

The mean inter-item correlations of the (sub)scales with combined items are in general lower than of (sub)scales with items all stated in the same direction. The alpha’s of the combined (sub)scales are all above.80 and for the overall scale even.95. Considering the reduction of scale length, lower alpha’s are to be expected with the smaller (sub)scales containing only positive or negative items. The reduction of alpha is however, especially for the negative items rather small.

## Discussion

In this paper we addressed a strategy that is adopted regularly with multi-item questionnaires, namely the use of reversed worded items. Many developers of questionnaires adopt this strategy with the intention of avoiding response bias, particularly acquiescence. We found evidence that this goal is not met. We also discussed an often unintended consequence of reversing some items, namely the debate arising in literature about the presence of two related but different conceptual features, like for example fitness and fatigue. We will discuss first the goal of reversing items and later the consequence. Response bias is a difficult phenomenon to detect as well as to prevent. We distinguished two types of response bias, response set and response style, and focused on characteristics of three types of response styles, that are often confused or overlooked in literature: acquiescence, inattention and confusion due to item verification difficulty. An acquiescent respondent has the tendency to affirm all statements. Pure acquiescence cannot be prevented by reversing half of the items. When all items assess fatigue and are worded in the same direction, an acquiescent person who is fatigued will receive a sumscore close to the true state. An acquiescent person who is not at all fatigued, however, will receive a biased sumscore. Thus, reversing half of the items will lead to a less biased sumscore for the latter respondent, but only at the cost of a more biased sumscore for the fatigue respondent. Therefore, reversing some items cannot be presumed to lead to better assessments in case of acquiescent respondents.

Inattention can relate to several different aspects of an item, like the specific content (is it about mental or physical fatigue), the time frame (is it about today, or the last week), but also the direction of the item (is it about being fatigue or not being fatigue) or the answer categories (do they range from always to never, or the other way around).

On closer examination, several characteristics can be distinguished, all covered by the concept inattention. Some respondents may be less precise in general. Others may become more inattentive in answering, depending on the length of the questionnaire and the extent to which the items resemble each other. And some may get frustrated by having to answer more or less the same items either in the same or opposite direction. Thus, in some cases extension of the questionnaire by more items, in the same or opposite direction, may work counterproductive.

Confusion is a response style dependent on the difficulty of the item and the cognitive strategies the respondent has to employ to give an answer that is in accordance with the true state.

We argued why reversing a portion of the items is an ineffective way of dealing with response bias. Reversing items by using negative particles or affixal morphemes will lead to increased difficulty, and thus more bias, without any clear advantage. Reversing some items by reversed worded items, may decrease item difficulty for those respondents that can agree with the reversed items, but at the same time will lead to more bias due to confusion for the other respondents, together with increased bias due to inattention for all respondents. In any case, acquiescence will not be avoided, at best detected. To a great extent, the confusion that is caused by reversing items, is due to the custom to present both original and reversed items mixed up.

Finally, we demonstrated that a particular instrument, the MFI-20, designed to prevent response bias using reverse wording of half the items, does not achieve this goal. The MFI-20 is a widely used instrument for reliable and valid assessment of fatigue in general and several types of fatigue. Results of the study raise questions whether the addition of ten reverse worded items, intended to prevent response bias, is justified. An added value of the negatively formulated items to the positive items, or vice versa, was not demonstrable. With respect to content of the items, the ten negatively and ten positively formulated items are measuring almost the same, if not exactly the same aspects of fatigue. Since the developers of the MFI explicitly stated that they added reverse worded items in order to tackle response bias, it would be useless to focus on any potential difference with respect to content or responsiveness of these ten items.

Instead of preventing response bias, the addition of ten reverse worded items appears to increase the risk of inattention and confusion. No intensive focus was put on potential subtle differences with respect to their content between two reverse worded items. Firstly, because the developers of the questionnaire explicitly stated that the purpose of adding reverse worded items was to prevent response bias. Secondly, any difference with respect to content should, if it is considered to be important, be assessed by items, all formulated in the same direction, in order to maximize opportunities to assess subtle differences and to avoid artifacts due to accidentally misreading.

The addition of ten reverse worded items did lead to slightly higher values of Cronbach’s alpha. This is however to be expected when scales are twice as long. With one exception, mean inter-item correlations decreased when adding ten reversed worded items, where and increase was to be expected, considering the reason for adding reversed items.

Considering the findings of Swain et al. [6], respondents seem to make less errors with items that reflect their experience or situation than with items that describe the opposite. Since this instrument is designed to measure fatigue, it will probably be used more often among persons with a certain level of fatigue. Therefore the negatively formulated items are to be preferred. The psychometric qualities of these ten items are acceptable, if not good.

A consequence of reversing items is the identification of two related but unipolar concepts where only one was intended. We expect some validity to the claim of unipolarity and thus two related concepts that are tapped by asking for both fitness and fatigue, positivism and negativism, happiness and sadness, being relaxed and nervous. However, we consider the emerging of these claims, originating from data-analysis, instead of from a theoretical position, a serious weakness. If distinguishing between two related but opposite concepts is truly relevant, it would be helpful to take precautionary actions to assess these concepts unambiguously. In accordance with Roszkowski and Soven [13], we suggest separate presentation of ordinary items and reversed worded items, instead of a list where these items are all mixed up.

Even when a multi-item questionnaire consists of items stated in the same direction, there are problems to be addressed that hamper an obvious relationship between the theoretical concept and the sumscore resulting from an addition of the itemscores [3]. Some aspects, commonly seen in multi-item instruments, that deserve to be addressed are:

1. Differences in item difficulty and their consequences for the interpretation of summed scores, a field that Item Response Theory is addressing.
2. Sometimes in the same questionnaire some aspects are addressed with more items than others, leading to an often unknown and implicit weighing of their contribution to the total score.
3. The rationale and consequences of using different answercategories for items that are supposed to belong to the same scale
4. The rationale and consequences of using both items asking for frequency and items asking for intensity.

All these phenomena deserve to be addressed. This discussion will be more fruitful if it is not obscured by effects resulting from reversed worded items.

In conclusion, we consider reversing items in order to prevent response bias a counterproductive strategy. Acquiescence cannot be prevented by reversing, and more errors will be made due to inattention or confusion. An instrument with all items formulated in the same direction and referring to the intended concept (i.e. fatigue or fitness, depression or happiness) is to be preferred. If a researcher is concerned about respondents missing subtle differences between the items, other strategies are to be considered.

It is surprising that reversing items, introduced several decades ago, is still predominant in many popular questionnaires. Discussion about the pros and cons of this phenomenon should be revived. Consider, on a rainy day, all cows in a pasture tending to stand facing in the same direction, with their back pointing from where the wind comes. We admit that one cow standing in the opposite direction, would be conspicuous immediately. Unfortunately items do not have a head and tail.
