# Correction: Ineffectiveness of Reverse Wording of Questionnaire Items: Let’s Learn from Cows in the Rain

**DOI:** 10.1371/annotation/af78b324-7b44-4f89-b932-e851fe04a8e5

**Published:** 2013-09-13

**Authors:** Eric van Sonderen, Robbert Sanderman, James C. Coyne

As a result of an error by PLOS ONE, an incorrect version of the article was typeset. Below is a link to a version of the article based on the correct manuscript.

Article: 
